# Supplementary figures and images for: Development of an omnidirectional rotating Compton camera for imaging 177Lu radioactive contamination
Source: PLoS One. 2025 Jun 23;20(6):e0325586. doi: 10.1371/journal.pone.0325586 (PMC12184998; doi:10.1371/journal.pone.0325586)

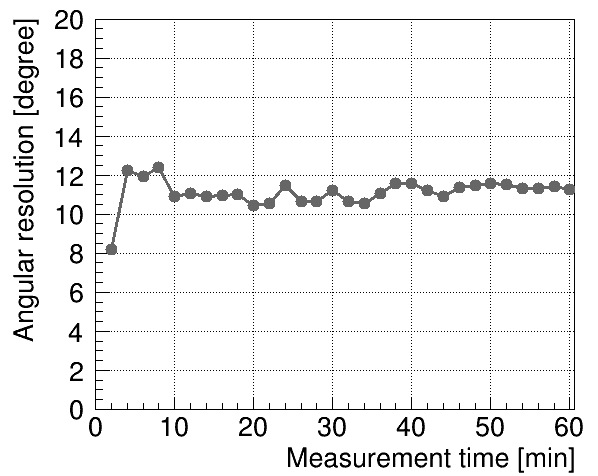

Supplement: S1 File — (ZIP) [file pone.0325586.s001.zip › S1_File/Figure11/Figure11_graph_AR.png]

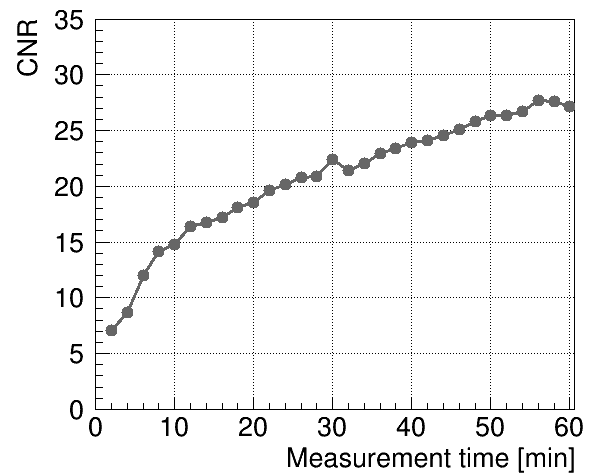

Supplement: S1 File — (ZIP) [file pone.0325586.s001.zip › S1_File/Figure11/Figure11_graph_cnr.png]

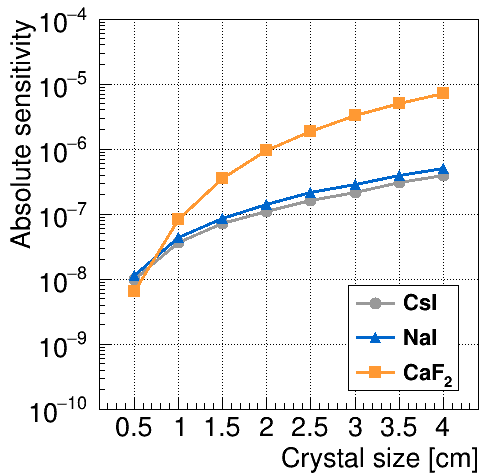

Supplement: S1 File — (ZIP) [file pone.0325586.s001.zip › S1_File/Figure5/Absolute_Sensitivity/113 keV/graph_AS_113keV.png]

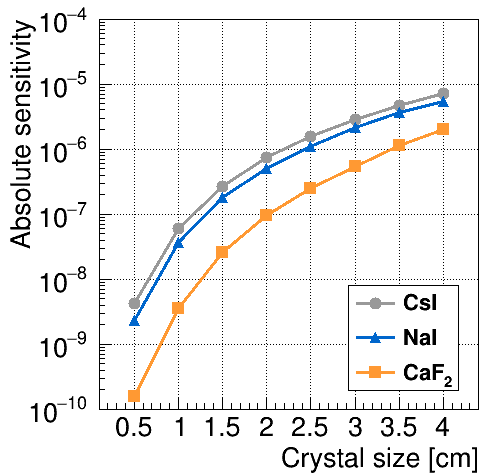

Supplement: S1 File — (ZIP) [file pone.0325586.s001.zip › S1_File/Figure5/Absolute_Sensitivity/1333keV/graph_AS_1333keV.png]

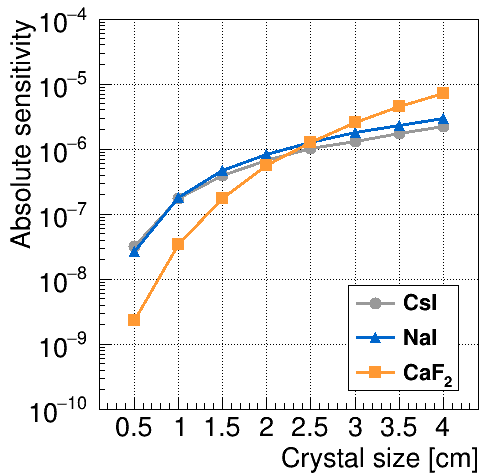

Supplement: S1 File — (ZIP) [file pone.0325586.s001.zip › S1_File/Figure5/Absolute_Sensitivity/208 keV/graph_AS_208keV.png]

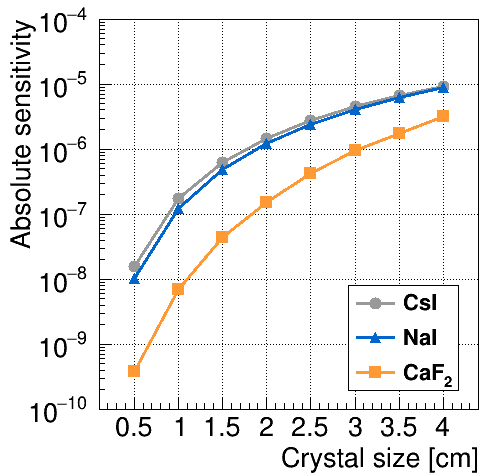

Supplement: S1 File — (ZIP) [file pone.0325586.s001.zip › S1_File/Figure5/Absolute_Sensitivity/511 keV/graph_AS_511keV.png]

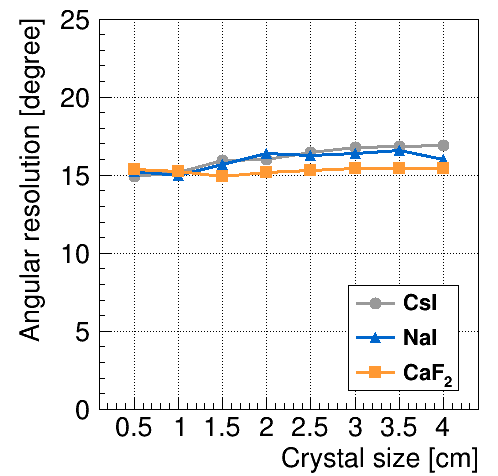

Supplement: S1 File — (ZIP) [file pone.0325586.s001.zip › S1_File/Figure5/Angular_Resolution/113 keV/graph_AR_113keV.png]

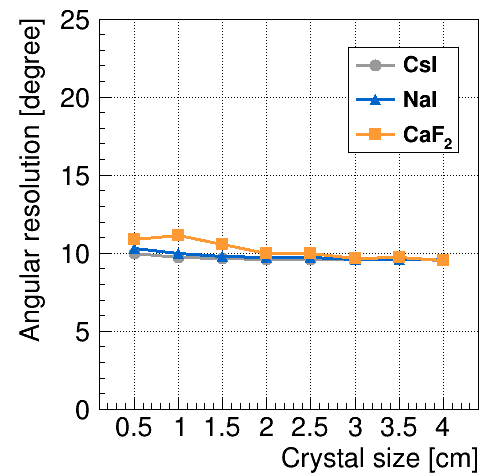

Supplement: S1 File — (ZIP) [file pone.0325586.s001.zip › S1_File/Figure5/Angular_Resolution/1333keV/graph_AR_1333keV.png]

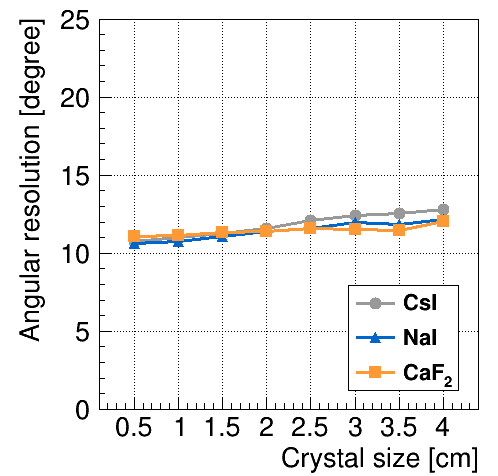

Supplement: S1 File — (ZIP) [file pone.0325586.s001.zip › S1_File/Figure5/Angular_Resolution/208 keV/graph_AR_208keV.png]

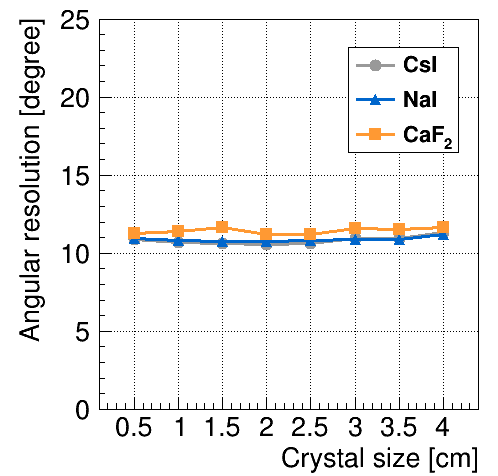

Supplement: S1 File — (ZIP) [file pone.0325586.s001.zip › S1_File/Figure5/Angular_Resolution/511 keV/graph_AR_511keV.png]

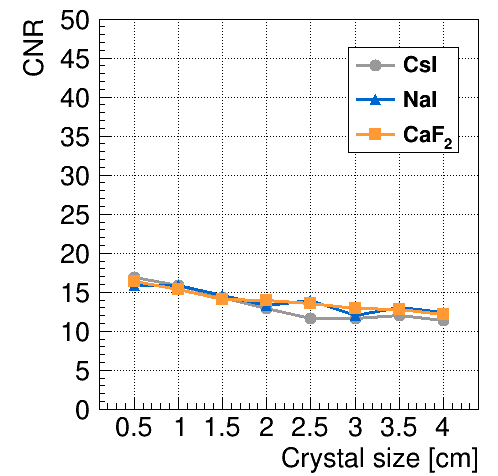

Supplement: S1 File — (ZIP) [file pone.0325586.s001.zip › S1_File/Figure5/CNR/113 keV/graph_CNR_113keV.png]

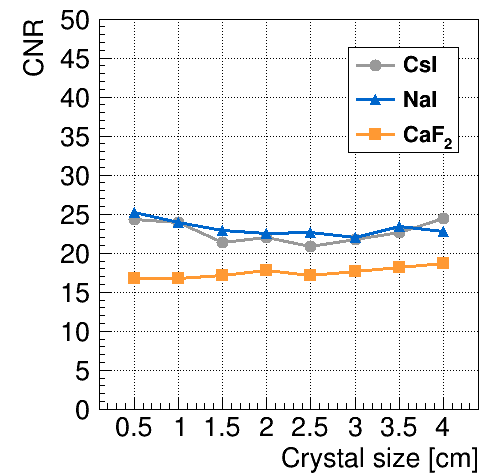

Supplement: S1 File — (ZIP) [file pone.0325586.s001.zip › S1_File/Figure5/CNR/1333keV/graph_CNR_1333keV.png]

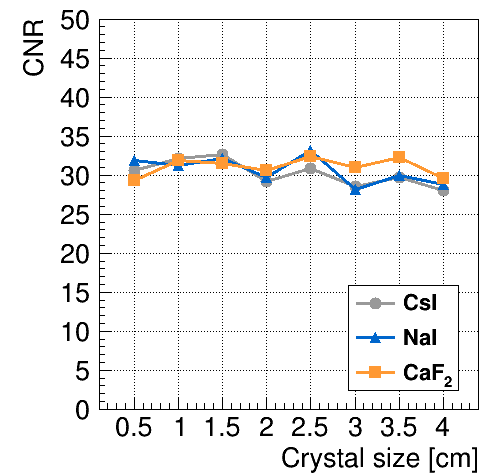

Supplement: S1 File — (ZIP) [file pone.0325586.s001.zip › S1_File/Figure5/CNR/208 keV/graph_CNR_208keV.png]

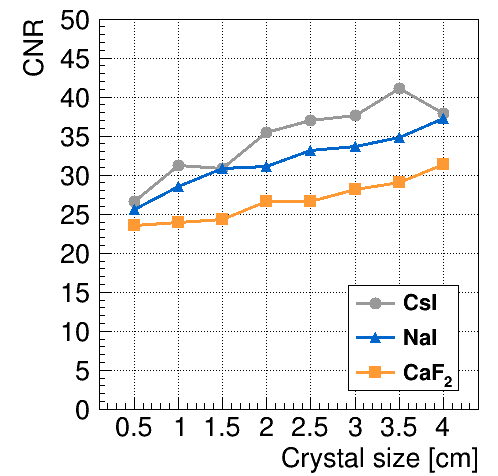

Supplement: S1 File — (ZIP) [file pone.0325586.s001.zip › S1_File/Figure5/CNR/511 keV/graph_CNR_511keV.png]

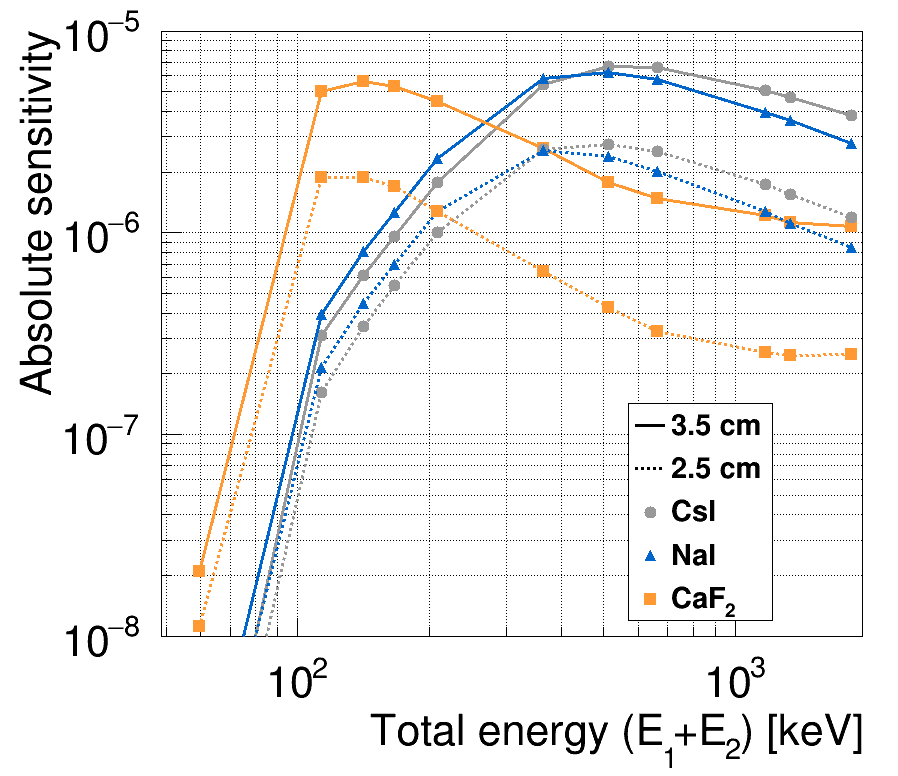

Supplement: S1 File — (ZIP) [file pone.0325586.s001.zip › S1_File/Figure6/Figure6.png]

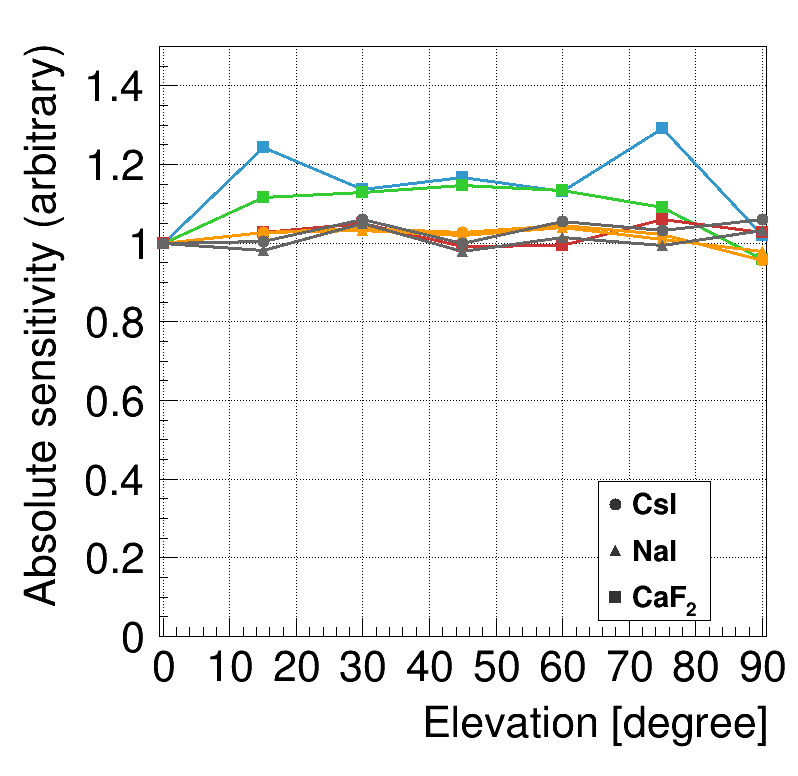

Supplement: S1 File — (ZIP) [file pone.0325586.s001.zip › S1_File/Figure7/Figure7.png]

## Slide 1
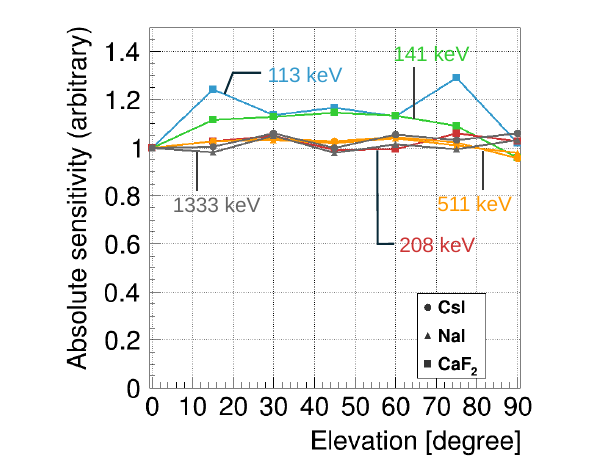

141 keV
113 keV
511 keV
1333 keV
208 keV

Supplement: S1 File — (ZIP) [file pone.0325586.s001.zip › S1_File/Figure7/Figure7.pptx]
