# Supplementary material for: Development of an omnidirectional rotating Compton camera for imaging 177Lu radioactive contamination
Source: PLoS One. 2025 Jun 23;20(6):e0325586. doi: 10.1371/journal.pone.0325586 (PMC12184998; doi:10.1371/journal.pone.0325586)
Supplement: Attachment — Submitted filename: Comments.docx [file pone.0325586.s002.docx]

The manuscript primarily discusses the research and development of an omnidirectional rotating Compton camera designed for imaging radioactive contamination from ^177^Lu. Monte Carlo simulations were conducted using Geant4 in a virtual setting to evaluate the detector's performance with various combinations of crystal types, sizes, and gamma-ray energies. The simulation findings indicated that a detector made of six 3.5-cubic-centimeter CaF_2_ crystals was the most suitable option. Following this, a prototype detector was created to visualize areas contaminated with ^177^Lu and ^99^mTc. Experimental results show that the rotating Compton camera technology excels in cost-effectiveness, sensitivity, and stability. The use of 208 keV gamma rays is ideal for imaging ^177^Lu, and this detector can simultaneously detect contamination from both ^177^Lu and ^99^mTc, which are frequently found in nuclear medicine facilities. This suggests its broad applicability in environmental radiation monitoring within such facilities. Its capability to quickly identify radioactive contamination could help minimize radiation exposure risks for medical staff and the public. However, prior to publication, the following issues need to be addressed:

In the course of developing the manuscript, what were the reasons for selecting the Monte Carlo simulation over alternative simulation techniques? Is the preference for this method attributable to its superior efficacy?

In the context of the Monte Carlo simulation, the selection of only three types of crystals—CsI, NaI, and CaF_2_—was made for specific experimental reasons, rather than an exhaustive evaluation of all available scintillator crystals. It is important to consider whether these three materials represent the optimal choices among scintillator crystals.

The manuscript indicates that the crystal size range utilized in the simulation spans from 0.5 to 4.0 cm. What rationale underpinned the selection of this specific range? What methodology was employed to ascertain this range? Furthermore, are there alternative size ranges that could potentially enhance the detector's performance?

The manuscript indicates that the detector is capable of concurrently visualizing the contamination of ^177^Lu and ^99^mTc. However, in practical applications, it is important to consider whether the simultaneous detection of these two contaminants may interfere with one another, potentially compromising the accuracy of the detection outcomes.

The manuscript discusses two methodologies for image reconstruction: the conventional technique and the image sharpening technique. In practical applications, which of these techniques is more prevalent? Which technique demonstrates greater utility? Additionally, have you contemplated the possibility of integrating these two approaches?

In the course of this research, what measures were implemented to ensure the accuracy and reliability of the experimental data? Were multiple repetitions of the experiments performed to mitigate potential errors? If so, what was the minimum error observed across these experiments?

The findings presented in the manuscript indicate that this detector has the potential to mitigate radiation exposure risks for both medical personnel and the general public. Consequently, what are the recommended protocols for the application of this detector in practical settings to effectively minimize these risks?

The manuscript does not provide an analysis of the detector's performance following extended usage. Could you clarify whether the detector's performance is expected to deteriorate over prolonged periods of use?
