# Supplementary material for: Development of an omnidirectional rotating Compton camera for imaging 177Lu radioactive contamination
Source: PLoS One. 2025 Jun 23;20(6):e0325586. doi: 10.1371/journal.pone.0325586 (PMC12184998; doi:10.1371/journal.pone.0325586)
Supplement: Attachment — Submitted filename: PLOS ONE Review reply - v10-1.docx [file pone.0325586.s003.docx]

----------------------------

Reply to reviewer #1:

----------------------------

N/A

----------------------------

Reply to reviewer #2:

----------------------------

Thank you very much for your comments. We have revised our manuscript accordingly. Revisions are indicated in blue font in the revised manuscript. We have also provided responses to your comments below (shown in blue fonts). Please note that some figure numbers have changed in the revised manuscript because of the added figure (Figure 11).

------------------------------------------------------------------------------------------------------------------------------------------------------------------------------(a) In the course of developing the manuscript, what were the reasons for selecting the Monte Carlo simulation over alternative simulation techniques? Is the preference for this method attributable to its superior efficacy?
Analytical approaches or statistical models can be considered alternative methods; however, interactions between radiation and matter are highly complex. Therefore, we used Monte Carlo simulation in this study. This simulation can flexibly model intricate geometrical conditions, making it particularly suitable for analyzing various crystal configurations. Based on this advantage, Monte Carlo simulation is the most applicable and practical approach for this study.

(b) In the context of the Monte Carlo simulation, the selection of only three types of crystals—CsI, NaI, and CaF_2_—was made for specific experimental reasons, rather than an exhaustive evaluation of all available scintillator crystals. It is important to consider whether these three materials represent the optimal choices among scintillator crystals.
We believe that the three materials (CsI, NaI, and CaF₂) are appropriate for this study.

In the Compton camera developed in this study, the angular resolution σ is defined by the root sum square of the three components derived through error propagation as follows:

σ = √(σ_E² + σ_Geo² + σ_Dop²)

Here, σ_E is the energy resolution of the scintillator crystals, σ_Geo is the geometrical factor determined by the crystal size and arrangement, and σ_Dop is the Doppler broadening limit. Among these variables, σ_Geo is the dominant factor in the Compton camera system. Because σ_Geo primarily depends on the geometrical configuration rather than the scintillator material, the type of scintillator has minimal influence on the overall angular resolution.

Furthermore, sensitivity at different gamma-ray energies, as shown in Figure 5, is influenced by the effective atomic number (Z_eff) of the scintillator. Therefore, we consider it reasonable to use three widely available and cost-effective scintillator crystals with different Z_eff values to assess the Compton camera system performance rather than evaluating all possible scintillator materials comprehensively.

(c) The manuscript indicates that the crystal size range utilized in the simulation spans from 0.5 to 4.0 cm. What rationale underpinned the selection of this specific range? What methodology was employed to ascertain this range? Furthermore, are there alternative size ranges that could potentially enhance the detector's performance?

As shown in Figure 5, the angular resolution (σ) and contrast-to-noise ratio (CNR) are independent of the crystal size when the interval between crystals is scaled proportional to the crystal size. Meanwhile, the crystal size considerably affects the absolute sensitivity of the detector. Although the absolute sensitivity improves with increasing the crystal size, it plateaus as the crystal size exceeds 4.0 cm. Therefore, we only report the results for crystal sizes up to 4.0 cm.

Notably, doubling the crystal size necessitates a proportional doubling of the intercrystal spacing, which, in turn, requires the doubling of the overall detector size. Such an increase in crystal size is impractical from the design and implementation perspectives. In addition, the cost of scintillator crystals increases proportionally with their volume. Therefore, extending the crystal size beyond 4.0 cm is not realistic in terms of detector design and cost-effectiveness.

(d) The manuscript indicates that the detector is capable of concurrently visualizing the contamination of ^177^Lu and ^99m^Tc. However, in practical applications, it is important to consider whether the simultaneous detection of these two contaminants may interfere with one another, potentially compromising the accuracy of the detection outcomes.

As described in the Discussion section (pages 13–14, lines 416–419), using a 208-keV gamma ray for ^177^Lu imaging ensures that there is no interference from gamma-ray events emitted from ^99m^Tc. Because the primary focus of this study is ^177^Lu imaging, this concern lies outside the main scope of the paper. However, as correctly noted, if ^99m^Tc imaging is considered, it may be affected by Compton scattering originating from ^177^Lu, as shown in the energy spectrum in Figure 14. The degree of this interference depends on the relative intensity of ^177^Lu compared to ^99m^Tc.

(e) The manuscript discusses two methodologies for image reconstruction: the conventional technique and the image sharpening technique. In practical applications, which of these techniques is more prevalent? Which technique demonstrates greater utility? Additionally, have you contemplated the possibility of integrating these two approaches?

As mentioned in the second paragraph of the Discussion section (page 14, lines 422–451), the Compton camera developed in this study supports conventional and image-sharpening techniques, allowing users to select the most appropriate method depending on the application and radioactive contamination environment. This flexibility makes the system suitable for cases where either rapid decontamination or a high angular resolution is prioritized. Each technique has advantages and limitations and should be considered an independent methodology. Furthermore, as discussed in the fourth paragraph of the Discussion section (page 15, lines 509–513), we consider the maximum likelihood expectation maximization (MLEM) method as an extension or integration of these two approaches. However, another reviewer has also asked about methodology. Therefore, we have described the two-step imaging strategy employed in this study from the perspective of the as low as reasonably achievable (ALARA) principle in the Discussion section of the revised manuscript (page 14, lines 451–460) as follows.

“This two-step imaging strategy can also be considered adequate from the perspective of the as low as reasonably achievable (ALARA) principles. The first step enables the rapid visualization of radioactive contamination, facilitating immediate decontamination while minimizing unnecessary radiation exposure to medical professionals and the public. The second step, which provides a high-resolution radioactivity distribution, identifies the direction of radioactive contamination in more detail. This minimizes the number and extent of decontamination operations required, thereby reducing the overall exposure and operational time. Accordingly, the proposed method offers a practical and realistic approach for radiation contamination management that aligns with the ALARA principles.”

(f) In the course of this research, what measures were implemented to ensure the accuracy and reliability of the experimental data? Were multiple repetitions of the experiments performed to mitigate potential errors? If so, what was the minimum error observed across these experiments?

Thank you for your comment. The energy resolution of the scintillator crystals used in this study is approximately 18.6% FWHM at 166 keV. Error arising due to the temperature dependence of the PMT, which is in the order of few percents, is sufficiently small relative to this energy resolution value and does not significantly affect the experimental results as a systematic error. Furthermore, before measurements, each counter is calibrated using a known energy source. For more details, please refer to Refs. [26] and [31]. In addition, to minimize the impact of fluctuations due to electronics or power instability, the power supply and preamplifier board are custom-developed. Multiple measurements are conducted in the laboratory using a ^139^Ce (166 keV, 174 kBq) source, and the results are reproducible within the statistical error range.

(g) The findings presented in the manuscript indicate that this detector has the potential to mitigate radiation exposure risks for both medical personnel and the general public. Consequently, what are the recommended protocols for the application of this detector in practical settings to effectively minimize these risks?

We have described an example of a protocol in the Discussion section (page 14, lines 422–460) as follows:

“The proposed detector employs two types of image reconstruction techniques: conventional and image sharpening techniques. The conventional technique enables rapid visualization, making it suitable for the immediate assessment of ^177^Lu-contaminated sites. Moreover, the image sharpening technique enhances the angular resolution, which is beneficial for precisely identifying the direction of radioactive contamination hotspots. Therefore, a two-step approach that uses both of these techniques would be preferable for application to actual ^177^Lu-contaminated sites...” However, practical applications, such as measurements using the detector in RI rooms or “special measures patient room,” have not yet been conducted. To further explore the development of appropriate protocols, an ethical review will be involved, and we plan to take the appropriate steps moving forward.

(h) The manuscript does not provide an analysis of the detector's performance following extended usage. Could you clarify whether the detector's performance is expected to deteriorate over prolonged periods of use?
In general, conventional detectors rely on calibration constants for energy determination, and these constants may change over extended periods of use. However, the developed Compton camera is designed to minimize such effects by allowing its calibration before each measurement. This is achieved by switching to a single-trigger mode, which enables entry into a dedicated calibration mode. Thus, performance fluctuations due to long-term usage are effectively suppressed.

As rightly pointed out, if the detector is used in high-dose-rate environments, such as medical accelerator facilities, where gamma-ray energies exceed 1 MeV, the scintillator can become radioactivated because of the presence of neutrons, increasing background and potential performance degradation. However, this study focuses on applications under nuclear medicine settings, which do not involve such high-energy or high-dose conditions. Therefore, performance degradation due to prolonged use is considered negligible under the usage conditions assumed in this study.

-----------------------------------------------------------------------

Furthermore, we have modified some parts by following the suggestions from another reviewer as described below.

(1) On page 9, lines 265–267, we added a statement to the part of “4. Discussion”:

“Notably, the simulation results shown above were consistent even when detailed simulations were performed using a physics list, such as LIVEMORE [35], which was specifically designed for low-energy gamma rays.”

(2) On page 9, lines 267–274, we added a statement to the part of “2.4. Simulation results”:

“Furthermore, as described in Section 2.1, the energy resolution σ(E) obtained by measuring CsI(Tl) was employed in simulations using NaI(Tl) and CaF₂(Eu). Nonetheless, the simulation results were consistent even when the energy resolution obtained by measuring 3.5 cm cubic CaF₂(Eu) coupled with a metal-packaged PMT (σ(*E*)=1.001*E*^0.4963, which is 10% larger than that for CsI(Tl)), was used. This implies that the angular resolution of the Compton camera used in this simulation is dominated by the geometrical factor, consistent with a previous report [32].”

(3) On pages 14–15, lines 470–481, we added a statement to the part of “4. Discussion”:

“However, because the composition and structure of the vial are not publicly disclosed, we performed Monte Carlo simulations to estimate the effect of self-absorption. In the simulation, the vial was assumed to be made of typical borosilicate glass ($\mathrm{SiO}_{2}:80\%$, $B_{2}O_{3}:13\%$, $\mathrm{Na}_{2}O:4\%$, $\mathrm{Al}_{2}O_{3}:3\%$) and was modeled as a cylindrical shape with an outer diameter of 31.5 mm, an inner diameter of 28.5 mm, and a height of 43 mm. To more accurately replicate the measurement conditions, we assumed a volume source filled with 25 mL of water instead of a point source. The self-absorption effect due to the liquid and glass vials was approximately 20%, indicating that the observed discrepancy can be attributed to this factor. Nevertheless, other potential factors, such as differences in the actual vial composition, calibration errors in the RI dose calibrator, and variations in the source positioning during measurements, should also be considered.”

(4) On page 11, lines 351–355, we added a statement to the part of “3.3.1. Measurement of ^177^Lu”:

“To further evaluate the time dependence of the reconstructed image, the angular resolution σ and CNR at 208 keV were analyzed every 2 min over a measurement period of 60 min (Fig. 11). The angular resolution σ rapidly improved within the first few minutes and gradually approached the value obtained from the 60-min measurement. In contrast, the CNR increased with measurement time.”

(5) On page 15, lines 481–490, we added a statement to the part of “4. Discussion”:

“Another potential cause of this discrepancy is the reproducibility of the simulation. A key difference between the simulation described in Section 2 and the detector setup described in Section 3 is the presence of the PMT. Assuming that the PMT is made of aluminum (density: 2.7 g/cm^3^), we conducted a Monte Carlo simulation with the PMTs arranged vertically, as shown in Fig 8(b). The absolute sensitivity of the 208 keV gamma rays, normalized to the absolute sensitivity at 0 degrees elevation (Fig 7), decreased by approximately 4% and 20% at elevation angles of 0 degrees and 90 degrees, respectively. The influence of the PMT on the absolute sensitivity of the simulation also contributed to the discrepancy in the detection efficiency.”

(6) On page 3, lines 73–80, we added a statement to the part of “1. Introduction”:

“As an example of a gamma-ray detector for environmental radiation imaging, Polaris-H was developed by the University of Michigan and commercialized by H3D [23]. It uses CdZnTe (CZT) and offers excellent energy resolution (ΔE/662 keV= 1.1% FWHM). This system can achieve relatively high sensitivity as a Compton camera at energies above 250 keV, making it particularly useful for identifying radioactive contamination in nuclear power plants. However, at energies below 250 keV, it operates as a coded-aperture gamma-ray camera, which limits its field of view and sensitivity.”

(7) On page 12, lines 383–387, we added a statement to the part of “3.3.1. Measurement of ^177^Lu”:

“To support this approach, we performed Monte Carlo simulations to evaluate the distance dependence of the absolute sensitivity. As a result, for source–detector distances 𝑟 ≥ 30 cm, the absolute sensitivity followed the inverse-square law (1/r^2^), which was within statistical uncertainty, validating the applicability of distance-based correction in this range.”

(8) On page 10, lines 322–326, we added a statement to the part of “3.2. Performance tests”:

“To prevent radioactive contamination in case of vial or syringe damage, the ^177^Lu and ^99m^Tc sources were each placed on trays lined with a filter paper during experiments. The operators wore gloves and used the personal dosimeters to ensure proper radiation protection. During measurements, the operators evacuated the controlled radiation area to minimize exposure as much as possible.”

----------------------------

Reply to reviewer #3:

----------------------------

Thank you very much for your comments. Below are our responses to your comments. We have also revised our manuscript based on your comments (revisions are shown in blue font). Please note that some figure numbers have changed in the revised manuscript because of the added figure (Figure 11).

------------------------------------------------------------------------------------------------------------------------------------------------------------------------------(a) The Geant4 simulation employs the FTFP-BERT physics list. Why was this specific model chosen for low-energy gamma-ray interactions (e.g., 113–208 keV)? Are there validated benchmarks for
In our Geant4 simulation, we select the standard FTFP-BERT physics list considering a balance between computational efficiency and general versatility. Although physics lists specifically designed for low-energy gamma rays (e.g., Penelope or Livermore) are available, they notably increase the computation time. In this study, there is no significant difference in the simulation results when using such low-energy-specific models.

The developed Compton camera reconstructs images based on energy deposited in the scintillator crystals. Preliminary comparisons reveal that the use of low-energy-specific physics models does not significantly affect the outcome.

To clarify this point, we have added the following statement in the Simulation results section of the revised manuscript (page 9, lines 265–267):

“Notably, the simulation results shown above were consistent even when detailed simulations were performed using a physics list, such as LIVEMORE [35], which was specifically designed for low-energy gamma rays.”

(b) ^177^Lu gamma-ray transport in CaF_2_ crystals?

We did not conduct any dedicated benchmark studies on ^177^Lu gamma-ray transport, specifically within CaF₂(Eu) crystals. However, as mentioned in our response to your comment (a), the impact of the scintillator crystal type on the image reconstruction results is minimal in this study, and its influence on the angular resolution is limited. Therefore, we consider that a benchmark specifically tailored to CaF₂(Eu) is not essential for the objectives of this research.

(c) The energy resolution formula σ(E)=0.5746E^0.5736 is adopted from a prior CsI(Tl) study. How does this generalize to CaF_2_(Eu), given differences in light yield and decay time (Table 2)? Provide experimental calibration data for the CaF_2_-PMT system.

We apologize for the poor explanation of this point. As you correctly pointed out, the energy resolution formula σ(E) = 0.5746 × E^0.5736 is adopted from our previous study using 3.5-cm cubic CsI(Tl). CaF₂(Eu) differs in light output and decay time and, ideally, should be treated separately. However, we find that the simulation results are consistent, even when the energy resolution obtained by measuring 3.5-cm cubic CaF_2_(Eu) coupled with a metal-packaged PMT (σ(E) = 1.001 × E^0.4963, which is 10% larger compared with that for CsI(Tl)), is used. This implies that the angular resolution of the Compton camera considered in this simulation is dominated by the geometrical factor. Furthermore, because this simulation considers only deposited energy in the scintillator, the simulation results are not affected by the difference in the decay time.

Our Compton camera has an angular resolution of approximately 11°, which is sufficient to determine the direction of radioactive contamination. The observed difference between the simulation and experimental results is within 1°, indicating good experimental consistency. Therefore, within the scope and objectives of this study, we consider that differences in the energy resolution among scintillators, such as CaF₂(Eu), CsI(Tl), and NaI(Tl), have only a limited effect on the overall performance.

If a design aiming for a high angular resolution is implemented, it will be necessary to define the energy resolution for each crystal type and size individually and revalidate the system accordingly.

We have added the following text to the Simulation results section of the revised manuscript (page 9, lines 265–274):

“Furthermore, as described in Section 2.1, the energy resolution σ(E) obtained by measuring CsI(Tl) was employed in simulations using NaI(Tl) and CaF₂(Eu). Nonetheless, the simulation results were consistent even when the energy resolution obtained by measuring 3.5 cm cubic CaF₂(Eu) coupled with a metal-packaged PMT (σ(*E*)=1.001*E*^0.4963, which is 10% larger than that for CsI(Tl)), was used. This implies that the angular resolution of the Compton camera used in this simulation is dominated by the geometrical factor, consistent with a previous report [32].”

(d) The discrepancy between simulated and measured detection efficiency (0.50 vs. 0.30 cps/MBq) is attributed to self-absorption in the vial. Include a Monte Carlo sub-study quantifying self-absorption effects for ^177^Lu in glass/liquid media to validate this claim.

Thank you for your suggestion. We have added the following discussion on the Monte Carlo simulation of the self-absorption effect of ^177^Lu in glass/liquid media in the Discussion section of the revised manuscript (pages 14–15, lines 470–481):

“However, because the composition and structure of the vial are not publicly disclosed, we performed Monte Carlo simulations to estimate the effect of self-absorption. In the simulation, the vial was assumed to be made of typical borosilicate glass ($\mathrm{SiO}_{2}:80\%$, $B_{2}O_{3}:13\%$, $\mathrm{Na}_{2}O:4\%$, $\mathrm{Al}_{2}O_{3}:3\%$) and was modeled as a cylindrical shape with an outer diameter of 31.5 mm, an inner diameter of 28.5 mm, and a height of 43 mm. To more accurately replicate the measurement conditions, we assumed a volume source filled with 25 mL of water instead of a point source. The self-absorption effect due to the liquid and glass vials was approximately 20%, indicating that the observed discrepancy can be attributed to this factor. Nevertheless, other potential factors, such as differences in the actual vial composition, calibration errors in the RI dose calibrator, and variations in the source positioning during measurements, should also be considered.”

(e) For simultaneous ^177^Lu and ^99m^Tc detection, the 113 keV and 141 keV peaks overlap. The 141 keV window is adjusted asymmetrically (-σ to +2σ). Provide a quantitative analysis of cross-contamination risks and how this adjustment minimizes false positives.

As mentioned in the manuscript, ^99m^Tc does not affect ^177^Lu imaging because 208 keV gamma rays should be adopted in ^177^Lu imaging. The quantitative consideration of ^99m^Tc imaging in the presence of ^177^Lu is beyond the scope of this paper. In the future, we will conduct long-term monitoring in a clinical setting after obtaining approval from the ethics committee.

(f) The sensitivity drops sharply at ±60° elevation due to PMT shielding (Figure 7). How does this angular dependency impact practical deployment in rooms with complex geometries (e.g., ceilings, floors)? Suggest design modifications to mitigate this limitation.

As you pointed out, our prototype detector shows a drop in sensitivity in directions with an elevation of ±60° or more (only 13.4% of all directions). This sensitivity drop in the upward direction with an elevation of 60° or more (6.7% of all directions) can be attributed to shielding by the PMT. Additionally, a sensitivity drop in the downward direction with an elevation of −60° or less (6.7% of all directions) is attributed to shielding by the PMT and electronics container. Sensitivity in these small directions can be reduced by replacing the PMT with a small photodetector, such as SiPM, and/or placing the electronics box away from the counters. These aspects will be considered in our next study. In addition, the imaging of complex, spread-out contamination at different distances in a room should be considered using 3D image reconstruction with stereo observation from multiple directions. These contents are beyond the scope of this paper and will be considered in our next study.

(g) The study mentions future work on maximum likelihood expectation maximization (MLEM). Why was filtered back-projection prioritized here? Include preliminary comparisons between the two methods to justify the current approach.

Filtered back projection (FBP) is a method that guarantees the analytical reconstruction of quantitative images, and image sharpening can be easily achieved by changing from a single Gaussian function to a triple Gaussian function, as mentioned in a previous study (Muraishi et al., JJAP 2020). Meanwhile, maximum likelihood expectation maximization (MLEM) is a completely different image reconstruction technique. This method has been employed in previous studies on Compton camera technology; however, it is less quantitative because the results vary depending on the number of iterations and shape of the source. Therefore, the application of this method to the proposed technique should be carefully considered, and we plan to report on this in a separate paper.

(h) Angular resolution and CNR values (e.g., σ = 12°, CNR = 28) are derived from 60-minute measurements. How do these metrics scale with shorter acquisition times (e.g., 1–10 minutes)? Provide error bars or confidence intervals for key parameters.

Thank you for your suggestion. We have added Figure 11 to the revised manuscript. We have also added the following text in Section 3.3.1 of the revised manuscript (page 11, lines 351–355):

“To further evaluate the time dependence of the reconstructed image, the angular resolution σ and CNR at 208 keV were analyzed every 2 min over a measurement period of 60 min (Fig. 11). The angular resolution σ rapidly improved within the first few minutes and gradually approached the value obtained from the 60-min measurement. In contrast, the CNR increased with measurement time.”

(i) Reducing crystal spacing from 10 cm to 7 cm reportedly doubles sensitivity but degrades angular resolution. Present a systematic trade-off analysis (sensitivity vs. resolution) to guide end-users in customizing the detector for specific applications.

In this study, we focus on monitoring environmental contamination caused by ^177^Lu, and we perform simulations by applying scaling to the crystals, assuming an angular resolution of approximately 11°. However, to thoroughly examine the details of your suggestion, simulations without scaling will be necessary, which is beyond the scope of this study. Therefore, we will address this issue in future studies. It is an excellent topic, and we will explore it in our next study.

(j) The compact metal-packaged PMTs introduce directional shielding. Quantify the gamma-ray attenuation caused by PMT materials (e.g., aluminum housing) using simulations or experimental measurements.

Thank you for your suggestion. To discuss Monte Carlo simulation quantifying gamma-ray attenuation caused by PMT materials, we have added the following text in Section 4 of the revised manuscript (page 15, lines 481–490):

“Another potential cause of this discrepancy is the reproducibility of the simulation. A key difference between the simulation described in Section 2 and the detector setup described in Section 3 is the presence of the PMT. Assuming that the PMT is made of aluminum (density: 2.7 g/cm^3^), we conducted a Monte Carlo simulation with the PMTs arranged vertically, as shown in Fig 8(b). The absolute sensitivity of the 208 keV gamma rays, normalized to the absolute sensitivity at 0 degrees elevation (Fig 7), decreased by approximately 4% and 20% at elevation angles of 0 degrees and 90 degrees, respectively. The influence of the PMT on the absolute sensitivity of the simulation also contributed to the discrepancy in the detection efficiency.”

(k) The prototype weighs 7.2 kg (excluding battery). Discuss its portability in real-world scenarios (e.g., handheld operation, mounting on robotic platforms). Include data on vibration resistance or thermal stability during rotation.

The prototype detector comprises a detection unit (1.8 kg) and electronics (5.4 kg), and we believe that a further weight reduction of the electronics is feasible. Although we do not evaluate vibration resistance or thermal stability during rotation, the intended use of the detector is within nuclear medicine facilities, where it remains stationary and operates in the stable temperature environment. Therefore, the impact of these factors is minimal. We will consider these aspects in future studies when we explore broader operational scenarios.

(l) How does the detector’s performance compare to commercial survey meters or other Compton cameras (e.g., Polaris-H, Si/CdTe-based systems) in terms of sensitivity, angular resolution, and operational speed?

The developed Compton camera is ready to use immediately after power-on, which is comparable to commercially available survey meters that use CsI(Tl) and/or NaI(Tl) scintillators coupled with photomultiplier tubes. Polaris-H has better energy and angular resolution than the proposed system and can achieve higher sensitivity as a Compton camera at energies above 250 keV, which is particularly useful for identifying radioactive contamination in nuclear power plants. However, at energies below 250 keV, it operates as a coded-aperture gamma-ray camera, which limits its field of view and sensitivity. Furthermore, because it uses CdZnTe (CZT), the system is very expensive ($170,000). Furthermore, materials used for preparing the proposed system cost only approximately $17,000. Therefore, when commercialized, the system will be much less expensive than Polaris-H. We have added the following text to the Introduction section of the revised manuscript (page 3, lines 73–80):

“As an example of a gamma-ray detector for environmental radiation imaging, Polaris-H was developed by the University of Michigan and commercialized by H3D [23]. It uses CdZnTe (CZT) and offers excellent energy resolution (ΔE/662 keV= 1.1% FWHM). This system can achieve relatively high sensitivity as a Compton camera at energies above 250 keV, making it particularly useful for identifying radioactive contamination in nuclear power plants. However, at energies below 250 keV, it operates as a coded-aperture gamma-ray camera, which limits its field of view and sensitivity.”

(m) The detector is proposed for high-dose environments (e.g., Fukushima). Has the radiation tolerance of CaF_2_(Eu) crystals and PMTs been tested under prolonged exposure (e.g., 1–10 mSv/h)?

In the Discussion section, we mention that imaging in the high-dose environment, such as Fukushima, can be further extended by customizing the type and size of the scintillation crystals. Although the current system includes a rotation function, we previously reported that imaging is feasible in the environment with dose rates up to 1 mSv/h using NaI(Tl) scintillator crystals with a diameter of 1/2 in (1.3 cm) in an earlier version of the detector.

Katagiri H, Satoh W, Enomoto R, Wakamatsu R, Watanabe T, Muraishi H, et al. Development of an all-sky gamma-ray Compton camera based on scintillators for high-dose environments. J Nucl Sci Technol. 2018;55(10):1172–9.

(n) The study claims semiquantitative estimation using peak values and absolute sensitivity. Clarify the mathematical framework for converting reconstructed intensity to activity (Bq) and validate it with multi-distance measurements.

Thank you very much for your valuable comment. In this study, we define the absolute sensitivity at a source-to-detector distance 𝑟 of 1 m. In addition, we investigate the variation in absolute sensitivity with changes in the source-to-detector distance 𝑟. Specifically, we perform Monte Carlo simulation to evaluate the distance dependence of the absolute sensitivity. For example, using a 3.5-cm cubic CaF_2_ crystal-based octahedral Compton camera and assessing the absolute sensitivity for a 208-keV gamma-ray source, we confirm that for 𝑟 ≥ 30 cm, absolute sensitivity follows the inverse-square law (1/r^2^) within statistical uncertainty. This result validates the application of distance-based correction in this range. We have added the following text in Section 3.3.1 of the revised manuscript (page 12, lines 383–387) to address the insufficient explanation in the original manuscript:

“To support this approach, we performed Monte Carlo simulations to evaluate the distance dependence of the absolute sensitivity. As a result, for source–detector distances 𝑟 ≥ 30 cm, the absolute sensitivity followed the inverse-square law (1/r^2^), which was within statistical uncertainty, validating the applicability of distance-based correction in this range.”

(o) The rotational motion "virtually" increases crystal count. Provide a mathematical proof or simulation demonstrating how rotation suppresses ghost artifacts across varying source geometries (e.g., multiple distributed sources).

Regarding the effect of ghost reduction by rotation, please refer to our previous study (Muraishi et al., JJAP 2020), which reveals that adding the rotation function can easily and significantly suppress ghosts, and it is possible to obtain shift-invariant reconstructed images when measuring two sources simultaneously. The evaluation of reconstructed images for more complex shapes (three and/or extended sources) is beyond the scope of this paper and will be considered in our next study.

(p) The two-step imaging strategy (conventional → sharpening) is proposed for contamination removal. How would this workflow integrate with existing radiation safety protocols (e.g., ALARA principles)? Include a case study or pilot deployment plan.

Thank you for your suggestion. We have added the following text in Section 4 of the revised manuscript (page 14, lines 451–460):

“This two-step imaging strategy can also be considered adequate from the perspective of the as low as reasonably achievable (ALARA) principles. The first step enables the rapid visualization of radioactive contamination, facilitating immediate decontamination while minimizing unnecessary radiation exposure to medical professionals and the public. The second step, which provides a high-resolution radioactivity distribution, identifies the direction of radioactive contamination in more detail. This minimizes the number and extent of decontamination operations required, thereby reducing the overall exposure and operational time. Accordingly, the proposed method offers a practical and realistic approach for radiation contamination management that aligns with the ALARA principles.”

(q) The detector’s sensitivity drops above 300 keV for CaF_2_(Eu) (Fig. 6). For broader applicability, discuss hybrid crystal configurations (e.g., CaF_2_ + CsI) and provide preliminary simulation results.

This is an interesting topic that we are currently investigating. However, it falls outside the scope of the present study, which focuses on imaging below 300 keV. In future studies, we will address hybrid crystal configurations and related simulation results.

(r) While ethical approval was deemed unnecessary, describe measures taken to ensure operator safety during experiments with unsealed ^177^Lu sources (e.g., contamination control, dosimetry monitoring).

Thank you very much for your helpful comment. We have added the following text in Section 3.2 of the revised manuscript (page 10, lines 322–326):

“To prevent radioactive contamination in case of vial or syringe damage, the ^177^Lu and ^99m^Tc sources were each placed on trays lined with a filter paper during experiments. The operators wore gloves and used the personal dosimeters to ensure proper radiation protection. During measurements, the operators evacuated the controlled radiation area to minimize exposure as much as possible.”

----------------------------

Reply to reviewer #4:

----------------------------

Thank you very much for your comments. Below are our responses to your comments. We have also revised our manuscript based on your comments (revisions are shown in blue font). Please note that some figure numbers have changed in the revised manuscript because of the added figure (Figure 11).

------------------------------------------------------------------------------------------------------------------------------------------------------------------------------(a) It is unclear if the designs for their Camera have been provided as supporting information, or the data used for their analysis been made openly available. These pieces of information are recommended prior to accepting this work for publication.

In this study, we develop a prototype octahedral Compton camera based on the simulation results. Therefore, some aspects such as the number and arrangement of crystals as well as structure of electronics remain open for further optimization. However, the key features of the octahedral crystal arrangement are illustrated in Figure 2, and electronics used are described in the main text.

Accordingly, we believe that additional data disclosure is not necessary at this stage.

(b) The europium-doped calcium fluoride crystals are sensitive to gamma radiation, however, the authors also mention other radiopharmaceuticals (Table 1), two of which are not gamma emitters (yttrium-90 and strontium-89). How easy would it be to replace the scintillator crystal with one that would detect the beta radiation from these radioisotopes, and would the performance characteristics be expected to be significantly different for beta as is shown in their work for gamma? It would seem as if a scintillator that was sensitive to beta emissions would be capable of detecting/imaging more radioisotopes that are listed on Table 1 than the gamma-sensitive crystal used in this work. Please comment.

The Compton camera is a technology that images incoming gamma rays. It cannot image incoming beta rays. The range of beta rays in the body is 1 mm or less, and they are almost entirely absorbed by the body. Therefore, a completely different technology is required to image the distribution of yttrium-90 and strontium-89 in the body.

(c) The authors note that the imaging performance of this camera is expected to be accurate to approximately 30 cm for a gamma source that is 1.5 meters away. Presumably, this accuracy could be improved by either moving the camera closer to the radiation source, or placing it in another location in the same room to “triangulate” the position of the radiation source. Can these points be addressed in the discussion?

We already addressed the possibility of three-dimensional image reconstruction using measurements from multiple directions in the final paragraph of '4. Discussion' (page 15, line 509-513). This approach aligns with the objective of improving the localization accuracy of the radiation source and is consistent with the direction of this study.

Improving spatial resolution by reducing the distance between the detector and the radiation source is also effective. However, this approach may increase radiation exposure to operators during actual use and, therefore, may not be compatible with the intended use of the system, which focuses on rapid detection in low-exposure environments.

Therefore, we consider that acquiring measurement data from multiple directions (triangulate) is a more practical and safer approach for improving positional accuracy.

(d) The authors note that some amount of gamma radiation is obscured by the PMT assembly and associated electronics (line 438 and Fig 8) and suggest that the sensitivity of the detection array could be improved by bringing the scintillators closer together, at a cost of angular resolution. Could there be a way to reconfigure the placement of the scintillators “on the fly” using a mechanically-translatable assembly (such as a Hoberman-type octahedral scaffold), or would the additional mechanical braces needed for such an assembly compromise the sensitivity?

Reconfiguring the scintillator arrangement “on the fly,” such as using a mechanically translatable assembly like that of a Hoberman-type octahedral scaffold, is worth exploring, depending on the measurement objectives. However, during reconfiguration, the complexity of the crystal arrangement can increase, and as you mentioned, the additional mechanical braces required for such an assembly may interfere with sensitivity. Therefore, the careful consideration of these trade-offs will be necessary to determine if improvement in sensitivity outweighs a potential reduction in angular resolution and overall performance. Another reviewer has also asked about the effect of PMT shielding. Therefore, we have discussed the Monte Carlo simulation of PMT shielding in the Discussion section of the revised manuscript (page 15, lines 481–490) as follows:

“Another potential cause of this discrepancy is the reproducibility of the simulation. A key difference between the simulation described in Section 2 and the detector setup described in Section 3 is the presence of the PMT. Assuming that the PMT is made of aluminum (density: 2.7 g/cm^3^), we conducted a Monte Carlo simulation with the PMTs arranged vertically, as shown in Fig 8(b). The absolute sensitivity of the 208 keV gamma rays, normalized to the absolute sensitivity at 0 degrees elevation (Fig 7), decreased by approximately 4% and 20% at elevation angles of 0 degrees and 90 degrees, respectively. The influence of the PMT on the absolute sensitivity of the simulation also contributed to the discrepancy in the detection efficiency.”

-----------------------------------------------------------------------

Furthermore, we have modified some parts by following the suggestions from another reviewer as described below.

(1) On page 9, lines 265–267, we added a statement to the part of “4. Discussion”:

“Notably, the simulation results shown above were consistent even when detailed simulations were performed using a physics list, such as LIVEMORE [35], which was specifically designed for low-energy gamma rays.”

(2) On page 9, lines 267–274, we added a statement to the part of “2.4. Simulation results”:

“Furthermore, as described in Section 2.1, the energy resolution σ(E) obtained by measuring CsI(Tl) was employed in simulations using NaI(Tl) and CaF₂(Eu). Nonetheless, the simulation results were consistent even when the energy resolution obtained by measuring 3.5 cm cubic CaF₂(Eu) coupled with a metal-packaged PMT (σ(*E*)=1.001*E*^0.4963, which is 10% larger than that for CsI(Tl)), was used. This implies that the angular resolution of the Compton camera used in this simulation is dominated by the geometrical factor, consistent with a previous report [32].”

(3) On pages 14–15, lines 470–481, we added a statement to the part of “4. Discussion”:

“However, because the composition and structure of the vial are not publicly disclosed, we performed Monte Carlo simulations to estimate the effect of self-absorption. In the simulation, the vial was assumed to be made of typical borosilicate glass ($\mathrm{SiO}_{2}:80\%$, $B_{2}O_{3}:13\%$, $\mathrm{Na}_{2}O:4\%$, $\mathrm{Al}_{2}O_{3}:3\%$) and was modeled as a cylindrical shape with an outer diameter of 31.5 mm, an inner diameter of 28.5 mm, and a height of 43 mm. To more accurately replicate the measurement conditions, we assumed a volume source filled with 25 mL of water instead of a point source. The self-absorption effect due to the liquid and glass vials was approximately 20%, indicating that the observed discrepancy can be attributed to this factor. Nevertheless, other potential factors, such as differences in the actual vial composition, calibration errors in the RI dose calibrator, and variations in the source positioning during measurements, should also be considered.”

(4) On page 11, lines 351–355, we added a statement to the part of “3.3.1. Measurement of ^177^Lu”:

“To further evaluate the time dependence of the reconstructed image, the angular resolution σ and CNR at 208 keV were analyzed every 2 min over a measurement period of 60 min (Fig. 11). The angular resolution σ rapidly improved within the first few minutes and gradually approached the value obtained from the 60-min measurement. In contrast, the CNR increased with measurement time.”

(5) On page 3, lines 73–80, we added a statement to the part of “1. Introduction”:

“As an example of a gamma-ray detector for environmental radiation imaging, Polaris-H was developed by the University of Michigan and commercialized by H3D [23]. It uses CdZnTe (CZT) and offers excellent energy resolution (ΔE/662 keV= 1.1% FWHM). This system can achieve relatively high sensitivity as a Compton camera at energies above 250 keV, making it particularly useful for identifying radioactive contamination in nuclear power plants. However, at energies below 250 keV, it operates as a coded-aperture gamma-ray camera, which limits its field of view and sensitivity.”

(6) On page 12, lines 383–387, we added a statement to the part of “3.3.1. Measurement of ^177^Lu”:

“To support this approach, we performed Monte Carlo simulations to evaluate the distance dependence of the absolute sensitivity. As a result, for source–detector distances 𝑟 ≥ 30 cm, the absolute sensitivity followed the inverse-square law (1/r^2^), which was within statistical uncertainty, validating the applicability of distance-based correction in this range.”

(7) On page 14, lines 451–460, we added a statement to the part of “4. Discussion”:

“This two-step imaging strategy can also be considered adequate from the perspective of the as low as reasonably achievable (ALARA) principles. The first step enables the rapid visualization of radioactive contamination, facilitating immediate decontamination while minimizing unnecessary radiation exposure to medical professionals and the public. The second step, which provides a high-resolution radioactivity distribution, identifies the direction of radioactive contamination in more detail. This minimizes the number and extent of decontamination operations required, thereby reducing the overall exposure and operational time. Accordingly, the proposed method offers a practical and realistic approach for radiation contamination management that aligns with the ALARA principles.”

(8) On page 10, lines 322–326, we added a statement to the part of “3.2. Performance tests”:

“To prevent radioactive contamination in case of vial or syringe damage, the ^177^Lu and ^99m^Tc sources were each placed on trays lined with a filter paper during experiments. The operators wore gloves and used the personal dosimeters to ensure proper radiation protection. During measurements, the operators evacuated the controlled radiation area to minimize exposure as much as possible.”
